# Integrated analysis of the molecular action of Vorinostat identifies epi-sensitised targets for combination therapy

**DOI:** 10.18632/oncotarget.18910

**Published:** 2017-07-01

**Authors:** Jodie F. Hay, Katrina Lappin, Fabio Liberante, Laura M. Kettyle, Kyle B. Matchett, Alexander Thompson, Ken I. Mills

**Affiliations:** ^1^ Blood Cancer Research Group, Centre for Cancer Research and Cell Biology, Queen's University Belfast, Belfast, UK; ^2^ Molecular Oncology Laboratory, MRC – University of Glasgow Centre for Virus Research, University of Glasgow, Glasgow, UK; ^3^ Ludwig Boltzmann Institute for Cancer Research, Vienna, Austria; ^4^ Haematopoietic Stem Cell Biology, MRC Molecular Haematology Unit, Weatherall Institute of Molecular Medicine, John Radcliffe Hospital, Oxford, UK; ^5^ Division of Cancer and Stem Cells, Centre for Biomolecular Sciences, University of Nottingham, Nottingham, UK

**Keywords:** acute myeloid leukaemia, Vorinostat, HDAC, epigenetics

## Abstract

Several histone deacetylase inhibitors including Vorinostat have received FDA approval for the treatment of haematological malignancies. However, data from these trials indicate that Vorinostat has limited efficacy as a monotherapy, prompting the need for rational design of combination therapies. A number of epi-sensitised pathways, including sonic hedgehog (SHH), were identified in AML cells by integration of global patterns of histone H3 lysine 9 (H3K9) acetylation with transcriptomic analysis following Vorinostat-treatment. Direct targeting of the SHH pathway with SANT-1, following Vorinostat induced epi-sensitisation, resulted in synergistic cell death of AML cells. In addition, xenograft studies demonstrated that combination therapy induced a marked reduction in leukemic burden compared to control or single agents. Together, the data supports epi-sensitisation as a potential component of the strategy for the rational development of combination therapies in AML.

## INTRODUCTION

Acute myeloid leukaemia (AML) is a heterogeneous clonal disorder of immature haematopoietic myeloid cells. For over 30 years, the standard induction treatment for AML has consisted of a combination of an Anthracycline, such as Daunorubicin or Idarubicin, with Cytarabine (Ara-C) [1]. With this regimen, a response rate of around 60-80% CR can be achieved in younger adults (18 to 60 years old). However, in older adults (> 60 years) outcome is poor as older patients do not tolerate intensive treatment as well as younger counterparts. As AML is considered a disease of the aging, with AML occurring most frequently in older patients, there is a need for novel less toxic treatments such as epigenetic therapies [1–3]. Histone deacetylase (HDAC) enzymes are key regulators of chromatin structure and post translational modifiers of both histones and non-histone proteins. HDAC enzymes promote a deacetylated state by removing acetyl groups from specific residues and function dynamically with histone acetyltransferases (HAT) that add acetyl groups back. Regulating transcriptional activation through chromatin conformation [4–6] and targeting HDACs is an attractive therapeutic option [7–9]. HDAC inhibitors (HDACi) offer varying selectivity towards HDACs, where they can inhibit more than one HDAC class (pan-) or single HDACs or classes (selective) [5]. Related toxicities for HDACi are low [7, 10], which may be particularly beneficial to vulnerable and elderly AML patients. Vorinostat (suberoylanilide hydroxamic acid, SAHA) is a hydroxamic acid derivative that inhibits the class I and II HDACs (*HDAC1, 2, 3* and *6*) and is only one of four HDAC inhibitors approved for clinical use by the FDA (Food and Drug Administration, US) [11, 12]. Vorinostat has proven efficacy in restoring aberrant acetylation in a number of cancers, including both solid and haematological malignancies [13]. It is in phase II clinical trials (NCT00305773, cliinicaltrials.gov) in AML as a monotherapy and has since entered clinical trials as part of a combination therapy with several different agents [14].

Non-histone proteins can also be modified by HDACs and subsequently HDACi inhibition of *HDAC6* also leads to acetylation of its substrate α-tubulin, inducing changes in cell motility [15]. Other forms of cell regulation affected by the acetylation of non-histone proteins as a result of HDAC inhibition with Vorinostat include cell proliferation (i.e. p53), DNA damage repair (i.e. Ku-70) and cell cycle (i.e. p21^WAF1/CIP1^) [6, 16–18]. However, the exact mechanism of how Vorinostat selectively targets cancer cells and achieves an effective clinical response in CTCL and other malignancies is not fully understood [10, 13].

A trial of Vorinostat as a monotherapy in advanced haematological malignancies identified a molecular response, histone H3 hyper-acetylation, in all patients. Of the 41 patients enrolled, 7 patients (17%) achieved complete response (CR), complete response with insufficient haematological recovery (CRi), or haematological improvement. Importantly, all 7 patients were diagnosed as having AML [19]. Although these results are encouraging, a larger proportion of AML patients were non-responsive or resistant to Vorinostat.

Better understanding of the mechanisms of action of epigenetic therapies are needed to establish their efficacy as either mono- or combination therapies [20]. In this study, we sought to further characterise the mechanisms of the HDACi Vorinostat through integrated ChIP-SEQ and gene expression analysis to identify potential novel, but rational, therapeutic combinations for Vorinostat.

## RESULTS

### Vorinostat exhibits potency in AML cell lines

**Figure 1:**
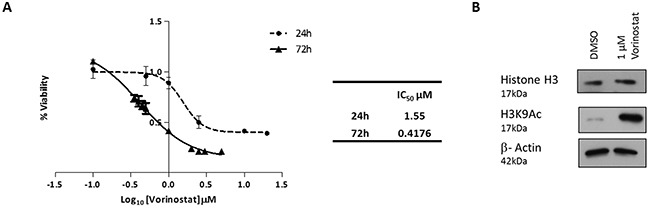
Vorinostat induced cell death and histone acetylation in AML cell lines **(A)** MTT cell proliferation assay derived dose response curves, for the OCI-AML3 AML cell line. OCI-AML3 cells were treated with Vorinostat for either 24 or 72 hours. Percentage of cell proliferation was calculated relative to DMSO (vehicle) control cells. IC_50_ values for the time points are shown in the table. **(B)** Western blot analysis confirmed that OCI-AML3 cells treated with 1 μM of Vorinostat for 24 hours, versus control conditions, was sufficient to inhibit HDACs as demonstrated by the acetylation of histone H3, and more specifically lysine 9 of H3.

Vorinostat exhibited greater potency at 72 hours (IC_50_ 0.42 μM) compared to the 24 hour time point (IC_50_ 1.55 μM; Figure 1A). A sub-IC_50_ dose of Vorinostat at 24 hours (1 μM) was sufficient to result in measurable acetylation of lysine 9 of histone H3 (Figure 1B). No changes in total histone H3 protein levels were observed. Therefore, the Vorinostat-treatment chosen for subsequent experiments was 1 μM for 24 hr. The OCI-AML3 cell line, which harbours a nucleophosmin (NPM1) mutation, exhibited a similar level of toxicity compared to HL-60, NB4 and U937 AML cell lines not carrying this mutation (Supplementary Figure 1A). Vorinostat induced toxicity was identified in HoxA9/Meis1 derived leukemic murine bone marrow but not in normal murine bone marrow (NBM) (Supplementary Figure 1B), an attractive aspect of HDACi especially in a disease of the elderly such as AML.

### Profiling Vorinostat induced changes in gene expression

**Figure 2:**
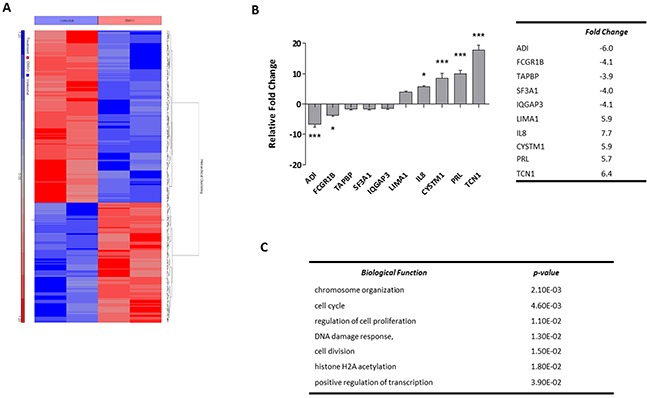
Profiling Vorinostat induced gene expression alterations in OCI-AML3 cells **(A)** Unsupervised hierarchical clustering of significantly differentially expressed genes in OCI-AML3 cells treated with either DMSO control conditions or 1 μM Vorinostat for 24 hours. The blue and red bars to the left of the heatmap show Vorinostat and DMSO samples respectively. On the heatmap, blue regions are indicative of low gene expression (142 genes down-regulated by Vorinostat), whereas high expression is represented by red regions (204 genes up-regulated by Vorinostat). **(B)** The top-5 most significantly up- and down-regulated genes were validated by RQ-PCR, and normalised to GAPDH. Data represents 3× technical replicates and 3× biological replicates. Significance, as calculated by two-way ANOVA, is denoted as * = P<0.05, ** = P<0.01 and *** = P<0.001. **(C)** The top biological pathways associated with significant alterations in gene expression. Of the 346 probe sets corresponding to the genes inputted, 313 were recognised by DAVID.

OCI-AML3 cells were treated for 24 hours with 1 μM Vorinostat and changes in gene expression examined using Affymetrix gene expression microarrays (Affymetrix™ GeneChip® Human Genome U133 Plus 2.0 Array). Possible confounding effects of DMSO treatment were controlled. Gene expression profiling and subsequent normalisation identified significantly differentially expressed genes, as expected due to it being an epigenetic modifying agent. To focus on prominent changes and pathways, the stringency for significance was set at a fold change of greater or less than 2-fold with an unadjusted p-value of <0.05. This identified 142 genes down-regulated by Vorinostat and 204 genes up-regulated (Figure 2A) (Supplementary Table 1). The top 5 up- and down-regulated differentially expressed genes (Figure 2B table) were validated by quantitative real-time PCR analysis (RQ-PCR). The RQ-PCR confirmed the directionality of the array findings which underestimated the extent of the fold-change tabulated in (Figure 2B). *In silico* functional analysis was undertaken using DAVID (Database for Annotation, Visualisation, and Integrated Discovery) (available from http://david.abcc.ncifcrf.gov/) which identified that the significantly enriched biological functional groups associated with the differential genes were chromosome organisation and cell cycle; DNA damage response and positive regulation of transcription (Figure 2C).

### H3K9 is acetylated with Vorinostat treatment

**Figure 3:**
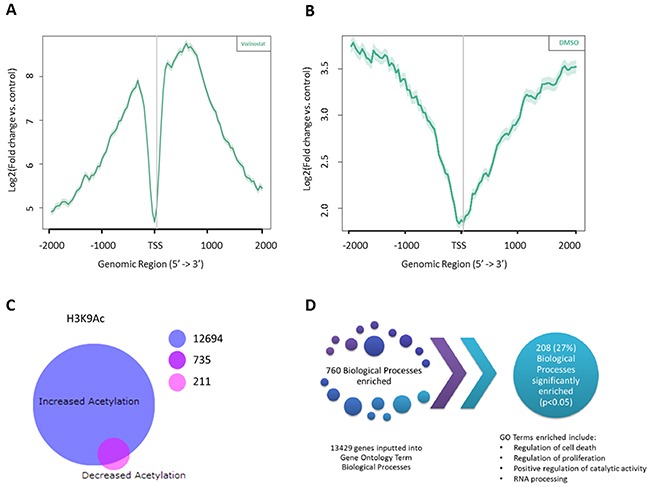
Identifying the global changes in the acetylation landscape in response to Vorinostat in AML cell lines with ChIP-SEQ Composite profiling of regions spanning the Transcription Start Sites (TSS) using histone H3 lysine 9 (H3K9) acetylation tag densities following treatment with either **(A)** Vorinostat or **(B)** DMSO vehicle control. The grey vertical line represents the TSS. **(C)** BioVenn diagram of Vorinostat induced acetylation and deacetylation of H3K9. Significant peaks (p-value (FDR) <0.05) and of good quality (Phred score >15) between the DMSO control and Vorinostat treated H3K9Ac datasets, were mapped to the closest Entrez Gene ID using HOMER, resulting in two genes lists; those significantly enriched for H3K9Ac and those associated with significantly less H3K9Ac. **(D)** Gene Ontology analysis of the 13429 genes associated with a significant increase in acetylation, following Vorinostat treatment, identified 208 biological processes that were significantly enriched.

Vorinostat is known to promote acetylation of a number of lysine residues along histone H3 and H4; however one of the most abundantly modified lysines is the activating histone mark lysine 9 on histone H3 (H3K9) [21]. Due to the distinct and accepted role of the acetylation of H3K9 as a marker of transcriptional activation [22, 23] it is therefore of interest to further the knowledge of Vorinostat within AML gene occupancy at the acetylated lysine 9 of histone H3 (Figure 1B). It was of interest to determine where H3K9 acetylation was enriched with regards to the transcriptional start site (TSS) and whether Vorinostat induces any changes to this. Chromatin from OCI-AML3 cells treated with 1 μM Vorinostat, or DMSO, for 24 hrs was isolated and DNA associated with H3K9 acetylation was immuno-precipitated. Template positive Ion Sphere Particles were run on the Ion PGM™ platform, the resulting data was indexed and aligned to hg19 using Bowtie2 [24, 25]. Histone H3K9 acetylation enriched peaks were identified using SICER [26] whilst ChIP Peak was used to develop composite profiles for the region spanning the TSS. These were plotted using the tag densities of the H3K9Ac samples and were expressed as a fold change relative to the input control for both Vorinostat (Figure 3A) and DMSO (Figure 3B) treatments. The DMSO sample had increased acetylation of H3K9 away from the TSS in up- or downstream by more than 1 kb. However, treatment with Vorinostat enriched H3K9Ac within 1 kb of the TSS. The intensity of this also increased, with the DMSO showing a peak tag density (log2 fold change) of approximately 3.75, whereas with Vorinostat induced acetylation of approximately 8.5. This reinforced that Vorinostat induces H3K9 acetylation, particularly with an enrichment close to the TSS (<1 kb).

### Vorinostat modulated pathways and networks

All significant ChIP-SEQ peaks (p-value (FDR) <0.05) of good quality (Phred score >15), between the DMSO control and Vorinostat treated H3K9Ac datasets, were mapped to the closest Entrez Gene ID using HOMER. This identified genes which were significantly enriched for H3K9Ac and those associated with significantly less H3K9Ac. Unsurprisingly 93% (13429 genes) of the Vorinostat induced differentially acetylated genes were only associated with H3K9Ac enrichment, whereas only 946 (7%) of genes showed a decrease in H3K9Ac. Interestingly, 735 (5%) genes demonstrated both acetylated and deacetylated H3K9Ac in response to Vorinostat treatment (Figure 3C).

Gene Ontology (GO) analysis was performed on the genes associated with an increased acetylation (13429 genes) and showed that cell death, cell cycle, cell proliferation and metabolism were significant (p<0.05). This resulted in 760 GO enriched pathways (Figure 3D). Surprisingly, of these 27% of these, 208 biological processes were found to be significant (p<0.05).

**Figure 4:**
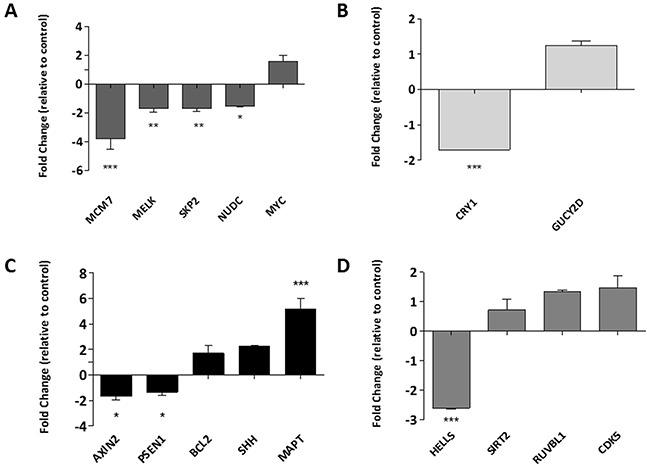
Identifying the biological processes associated with changes in Histone H3K9 acetylation Gene lists associated with specific biological functions of interest, those which feature in the functional annotation of the ChIP-SEQ dataset were compiled and subjected to network analysis. The genes which were identified as central to these networks were selected for further validation by RQ-PCR in networks associated to **(A)** cell proliferation, **(B)** metabolism, **(C)** cell death and **(D)** cell cycle. Data is presented as a fold change relative to the DMSO control, whereby any genes below the x-axis are down-regulated and those above, up-regulated. Experiment was carried out in a biological replicate of three. Significance, as calculated by two-way ANOVA, is denoted as * = P<0.05, ** = P<0.01 and *** = P<0.001.

Further analysis using the online protein mapping tool STRING identified four networks (Supplementary Figure 2). Several genes from these networks associated with cell proliferation, metabolism, cell death, and cell cycle were selected for further analysis (Figure 4A–4D respectively), although only 50% of these showed a correlation between increased gene acetylation and increased expression.

### Integrated analysis identifies genes associated with induced acetylation and increased expression

**Figure 5:**
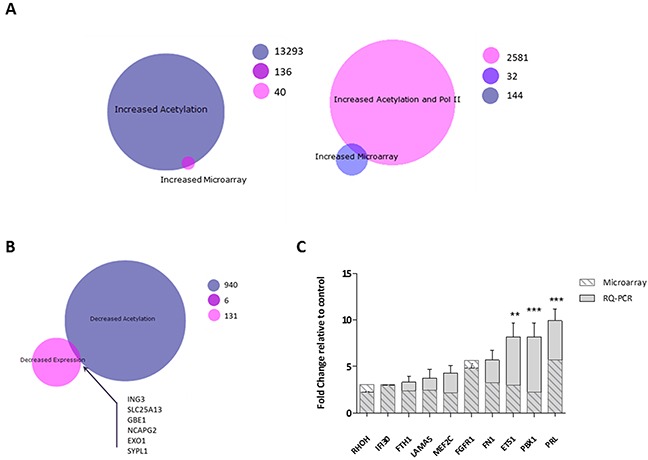
Integrative analysis of Vorinostat induced gene expression changes and alterations in the H3K9 acetylation landscape BioVenn diagrams of to determine overlaps between **(A)** genes with an increase in expression and an increased H3K9Ac binding (left) and genes with an increase in expression and genes associated with an increase in H3K9Ac and Pol II binding (right) **(B)** genes with a decreased expression and a decrease in H3K9Ac binding. Genes in the overlaps, 136 for the increased acetylation/increased expression and 6 for the decreased acetylation/decreased expression, were mapped for protein-protein interactions in STRING, with **(C)** expression of genes central for the networked measured by RQ-PCR. Experiment was carried out in a biological replicate of three. Significance, as calculated by two-way ANOVA, is denoted as * = P<0.05, ** = P<0.01 and *** = P<0.001.

Microarray data and enriched genes form the ChIP-SEQ study were integrated to provide a more comprehensive view of mechanism of action of Vorinostat. The 176 genes identified as showing up-regulation from the transcriptomic analysis were overlapped with the 2613 genes associated with a Vorinostat induced increase in H3K9Ac binding and Pol II binding (a marker of transcriptional activity). This resulted in only an overlap of 32 genes (Figure 5A). However, when Pol II was removed from data filtering, 136 genes were found to have increased gene expression and increased acetylation, suggesting that H3K9Ac is not only a marker of pre-transcriptional signalling, but also of transcribed genes. Comparison of genes associated with a decrease in gene expression (137) and a decrease in H3K9Ac enrichment (946), identified 6 genes in the overlap (Figure 5B). Further protein-protein interactions analysis in STRING resulted in identification of a network of 48 linked genes associated with both increased acetylation and expression (Supplementary Figure 3). Ten genes were selected and validated by RQ-PCR as being up-regulated following Vorinostat treatment (Figure 5C).

### SHH is an epigenetic induced therapeutic target

**Figure 6:**
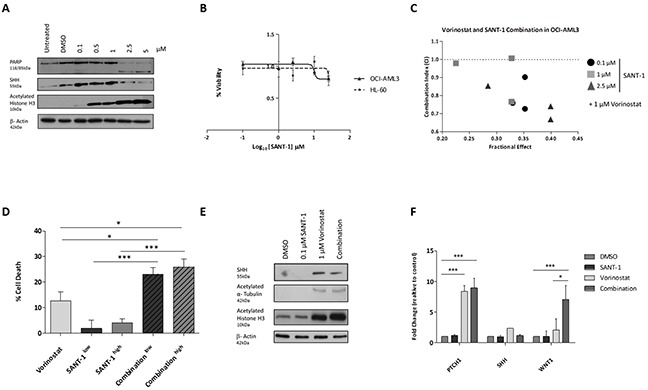
The sonic hedgehog signalling pathway is a therapeutic target for combination therapy SHH expression was measured in **(A)** OCI-AML3 cells treated with Vorinostat or DMSO for 24 hrs. Equal loading was confirmed with β-Actin. **(B)** SANT-1 dose response in AML cell lines after 24 hours. Cell viability was calculated relative to the DMSO control. **(C)** Combination Index (CI) for 24 hour treatments of 1 μM Vorinostat with a concentration of 0.1, 1.0 or 2.5 μM of SANT-1. Data represents a biological replicate of 3. Values shown indicate that most combinations are synergistic (CI < 1). **(D)** Percentage cell death of OCI-AML3 cells was measured using the CellTiter-Glo® viability assay following 24 hours treatment with either sequential treatment of 1 μM Vorinostat with low (0.1 μM) or high dose (2.5 μM) SANT-1, or single agents. Data represents a biological replicate of 3, with data presented relative to the DMSO control (0% cell death). **(E)** Protein expression of SHH was measured following 24 hours treated with the four treatment strategies in OCI-AML3 cells. Equal loading was confirmed with β-Actin. **(F)** *SHH* gene expression was analysed with RQ-PCR. Experiment was carried out in a biological replicate of three. Significance, as calculated by two-way ANOVA, is denoted as * = P<0.05, ** = P<0.01 and *** = P<0.001.

Our analysis identified a number of antagonistic up-regulated genes in response to epigenetic priming of the genome by Vorinostat. However, these epigenetic induced targets provide an attractive platform for combination therapy. SHH has been reported to be associated with adverse effects in leukaemia [27]. The modest upregulation of *SHH* mRNA expression following Vorinostat treatment (Figure 4C), was verified at the protein level in a dose dependent manner in two AML cell lines, OCI-AML3 (Figure 6A) and HL-60 (Supplementary Figure 4A). As proof of principal, we tested the tested the potential of inhibiting SHH as a rational partner for combination therapy. SANT-1, a high affinity antagonist of smoothened (SMOH), was ineffective at inducing cell death as a single agent in the OCI-AML3 and HL-60 cell line models (Figure 6B). To assess whether the efficacy of SANT-1 could be improved by combining it with Vorinostat, varying concentrations of SANT-1 were used with a 1 μM dose of Vorinostat, and drug-dose response [28] measured to establish combination indices (CIs) (Figure 6C). The combination index demonstrated potent synergy across most concentrations used with CIs indices <1. Although a concentration of SANT-1 of 2.5 μM had the highest synergy with 1 μM Vorinostat, a lower concentration of 0.1 μM of SANT-1 was also used for further studies to establish a minimum effective dose with limited potential toxicity for future extension into pre-clinical models.

To confirm whether the combination of Vorinostat and SANT-1 was efficacious, OCI-AML3 cells were treated for 24 hours with sequential treatment of 1 μM Vorinostat and low dose (0.1 μM) or high dose (2.5 μM) SANT-1, or as single agents. The extent of cell death, measured by the CellTiter-Glo® Luminescent Cell Viability Assay, significantly increased with both low and high dose combination treatment (∼25% and ∼27.5%) compared to Vorinostat only treatment (∼12%) which was the next most potent treatment (Figure 6D). This trend was also observed in the HL-60 cell line, demonstrating a significant increase in cell death high dose SANT-1 with Vorinostat opposed to Vorinostat treatment alone (Supplementary Figure 4B). Cell cycle analysis of combining a concentration of 1 μM of Vorinostat with low dose SANT-1 (0.1 μM) showed a significant increase in SubG1 (p<0.0005) compared to the DMSO treated profile in the OCI-AML3 cell line (Supplementary Figure 4C). Limited changes in SubG1 populations were evident between combination and Vorinostat as a single agent. However, protein analysis identified a modest reduction in SHH with OCI-AML3 cells treated with the Vorinostat/SANT-1 combination than those treated with Vorinostat alone (Figure 6E). Interestingly the combination treatment induced a marked increase in histone H3 acetylation over Vorinostat alone. Gene expression analysis revealed a repression of *SHH* following low dose combination treatment, returning expression to basal levels compared to Vorinostat alone (Figure 6F). In response to this we looked at the downstream regulator of the sonic hedgehog pathway, *PTCH1*, and *WNT1*, a sonic hedgehog crosstalk signalling pathway. In response to the combination treatment *PTCH1* was induced to significant levels compared to the DMSO vehicle control (P<0.001). *WNT1* mRNA expression was significantly increased with combination therapy compared to the DMSO control (P<0.01), and elevated to a greater extent (P<0.05) than the Vorinostat only treatment.

### Reduced leukaemia burden in Vorinostat-SANT-1 treated recipient mice

**Figure 7:**
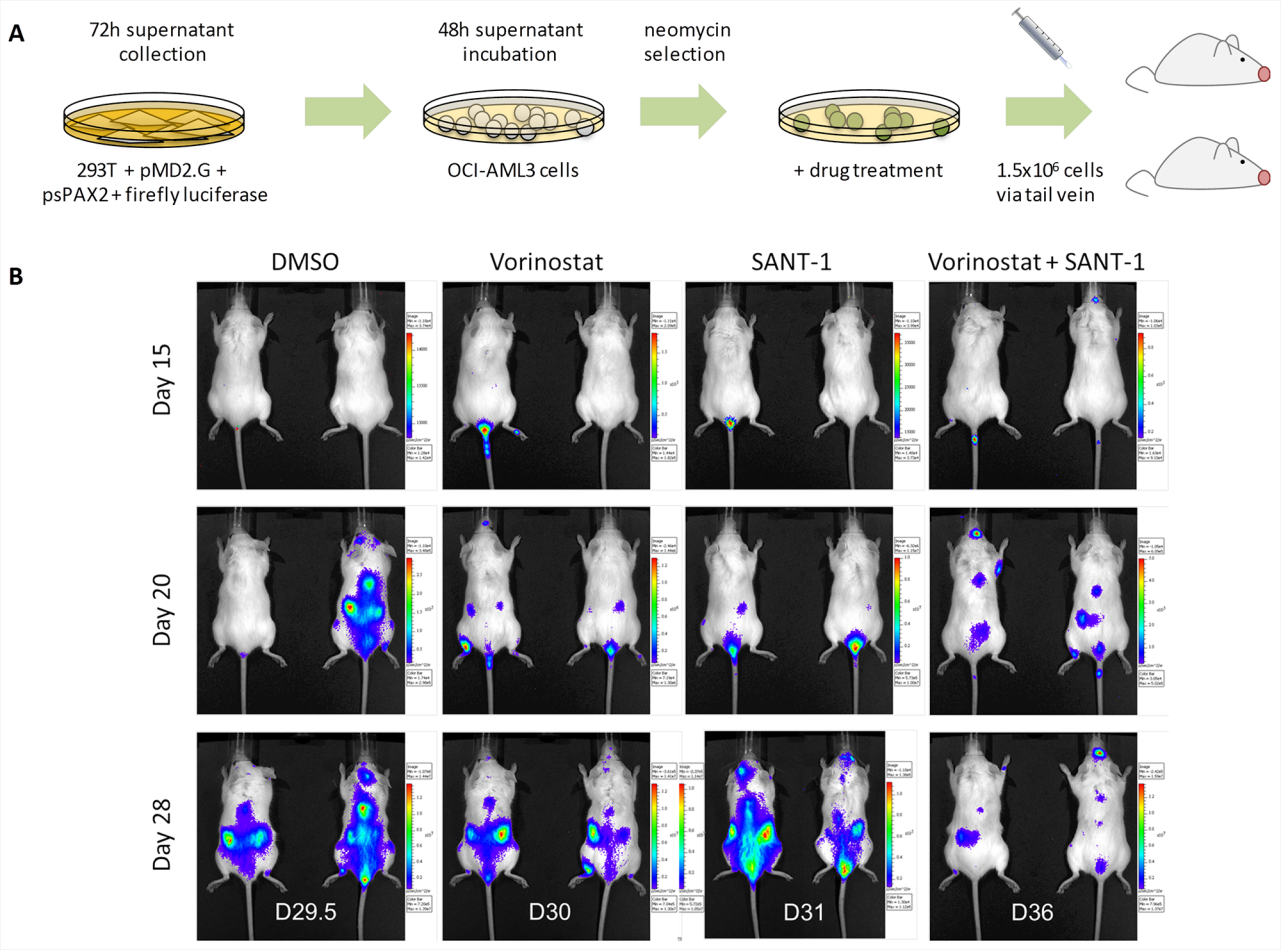
Vorinostat and SANT-1 combination reduces cancer burden in an AML xenograft model **(A)** Outline experimental design for AML xenografts derived from OCI-AML3 cells transduced with firefly luciferase, selected, and drug treated for 24 hours with DMSO vehicle control, 1 μM Vorinostat, 2.5 μM of SANT-1 or combination treatment. Treated cells were transplanted into NSG mice (n=2) via tail vein. **(B)** Mice were imaged at days 15, 20 and 28 to assess cancer burden. Mice were sacrificed at the first sign of disease and survival extension was assessed. Average survival post-transplant for each treatment arm were as follows: D29.5 (DMSO), D30 (Vorinostat), D31 (SANT-1) and D36 (Vorinostat and SANT-1).

OCI-AML3 cells which were retro-virally infected with a firefly luciferase marker, selected and subsequently treated for 24 hours with four treatment arms namely: DMSO, 1 μM Vorinostat, 2.5 μM SANT-1 or 1 μM Vorinostat + 2.5 μM SANT-1 (Figure 7A). Two NOD.Cg-Prkdc^scid^ Il2rgtm^1Wjl^/SzJ (NSG) mice for each treatment arm were injected with 1.5×10^6^ cells via tail vein. Leukaemia burden was followed via serial bioluminescent imaging at days 15, 20 and 28. At day 20, all mice from all treatment arms have evidence of leukaemia burden (Figure 7B). At day 28, no reduction in leukaemia burden was observed in mice receiving the DMSO vehicle control or SANT-1 treated cells. Recipients of the Vorinostat treated cells showed a modest reduction in leukaemia growth compared to control or SANT-1 treatment arms. However, the most striking reduction in leukaemia burden was seen with the Vorinostat and SANT-1 combination treatment at day 28. Combination therapy also extended life by an average of 6 days compared to Vorinostat, the next most effective treatment.

## DISCUSSION

It is well established that both genetics and epigenetics play a part in the development of cancer [29–31]. Epigenetic gene regulation involves heritable changes to the chromatin which do not change the DNA sequence, which include DNA methylation and chromatin remodelling [32]. The epigenetic landscape and regulation of AML has been shown to be aberrant and therefore provides attractive therapeutic targets [29, 30, 33, 34]. However, the characterisation of these epigenetic modifiers, including Vorinostat, remains incomplete. With resistance to Vorinostat evident from clinical trials, and insufficient activity to warrant using Vorinostat as a monotherapy, a better understanding of the drug is crucial [19, 31, 35]. This cell-line based study aimed to understand the mechanisms of Vorinostat on cell death, ultimately providing a more comprehensive analysis of Vorinostat by combining gene expression data with genes identified as being associated with acetylated H3K9. From this, the functional characterisation of the consequence of treating a cell with the HDAC inhibitor Vorinostat has been elucidated, including the identification of several, epigenetic primed or epigenetically induced therapeutic targets for a more rational approach to combination therapies [36, 37].

The regulatory processes of HDAC inhibition were underlined with transcriptome analysis of the OCI-AML3 myeloid cell line in the presence and absence of Vorinostat. The Vorinostat signature we observed was significantly enriched for regulators of the cell cycle, cell proliferation and the DNA damage response. Our analysis into the global impact Vorinostat has on histone H3K9 acetylation, identified enrichment around gene promoters and downstream of the TSS of active genes following treatment [38]. It is suggested that the acetylation of promoter regions surrounding the TSS, may provide stability for the binding of other chromatin remodelling factors and facilitate in RNA Pol II binding [38, 39].

Furthermore, our data reinforced the broad biological functions altered in the HDAC equilibrium. Of the 760 pathways enriched, 27% were enriched in cellular functions including key biological process such as proliferation, cell cycle and cell death. The involvement of the latter function fits well with our previously published work where we reported that Vorinostat can potently down-regulate the anti-apoptotic protein C-FLIP in AML [40]. As acetylation islands are reported to predict functional drivers, the integration of transcriptomics data and acetylation sequencing is a logical step in driver discovery [38, 41].

By priming, or epi-sensitising, cells with the epigenetic modifier Vorinostat, possible induced therapeutic targets could be identified through transcriptomic analysis and deep sequencing. Our analysis process has identified that, amongst others, the sonic hedgehog signalling pathway may be a potential, and detrimental, target due its pathway activation. *SHH* is usually inactivated in adult human tissues [42] but aberrant activation of the hedgehog pathway has been reported in a number of cancers [43–46], with it being noted as a driver in medulloblastoma [47]. Furthermore in a haematological setting, the activation of components of the pathway have aided in CML progression [27] and has been cited to promote the self-renewal capacity of human leukaemia cells and has a role in malignant potential in myeloid leukaemia [48, 49]. The use of the SHH inhibitor SANT-1 has shown encouraging results as a monotherapy or in combination with other agents [50, 51]. Whilst SANT-1 demonstrated little efficacy as a single agent in our study it proved to have high synergy when combined with Vorinostat both *in vitro* and in an *in vivo* xenograft model. Strikingly, mice injected with a single bolus of combination therapy treated cells exhibited a profound reduction in leukaemia burden compared to other treatment arms.

The concept of epi-sensitisation, epigenetically priming cancers prior to chemotherapy, is becoming more accepted, with growing evidence that epigenetic agents are altering the transcriptional potential of cancer cells [36, 52, 53]. In addition, clinical trials are highlighting some lower than expected response rates, relapse and evidence of drug resistance a persisting problem. In a clinical setting epi-sensitisation, in combination with rational therapeutic agents, may provide greater response rates.

## MATERIALS AND METHODS

### Cell lines

The AML cell lines HL-60, U937, NB4 and OCI-AML3 (DSMZ, Berlin, Germany) were maintained in RPMI 1640 media with L-Glutamine, supplemented with 10% Fetal Calf Serum (FCS) and 5% Penicillin/Streptomycin (Penicillin 100 μg/μl: Streptomycin 100 μg/μl) (Thermo Fisher Scientific, Paisley, UK).

### Trypan blue exclusion assay

In brief, 20 μl of cell suspension was mixed with an equal volume of Trypan Blue dye (Invitrogen Life Technologies, Paisley, UK) and incubated for 5 min. Cells were counted using a haemocytometer.

### MTT cell viability assay

MTT solution was prepared following manufacturer's instructions. In brief, 100 μl of cells with varying drug concentration, was dispensed in triplicate into a 96 well plate for both 24 and 72 hour time points, and 10 μl of prepared MTT solution (5 mg/ml) (Sigma-Aldrich Company Ltd., Germany) added. Plates were incubated for 3 hours at 37°C, and centrifuged at 200 X g for 5 minute. Supernatant was removed and the pellet was resuspended in 200 μl of DMSO before reading the plates at 590 nm using the Tecan Plate Reader, and Magellan software.

### CellTiter-Glo® luminescent cell viability assay and combination indices (CI)

The CellTiter-Glo® assay reagents were prepared as per the manufacturers recommended instructions. Into a white 96-well plate (Nunc™, Thermo Fischer Scientific), 25 μl of cell suspension was added along with 25 μl of prepared CellTiter-Glo® reagent. Plates were incubated for 30 minutes at room temperature. Luciferase activity was determined using a luminometer. To measure the dose–effect relationship of each drug and its combination and to determine synergy, CIs were calculated using the Calcusyn software package (BioSoft). CI values less than 1 are considered synergistic.

### RNA extraction and microarray

Total RNA was isolated by RNeasy kit (Qiagen, Manchester, UK). Eluted RNA was quantified using the Nanodrop ND-1000 Spectrophotometer (Thermo Scientific, Delaware, USA). Screening of total RNA samples against the Affymetrix™ GeneChip® Human Genome U133 Plus 2.0 Array followed the Microarray Sample Preparation Protocol as developed by Roche (Roche Diagnostics Ltd, West Sussex, UK) as part of the MILE study (23). The generated CEL files were saved and imported into Partek Genomics Suite 6.0 for bioinformatics analysis. Microarray data are available at the Gene Expression Omnibus (GEO) repository, accession number GSE86445. Matched samples were used for both the microarray and ChIP-SEQ.

### Quantitative real-time PCR

cDNA was prepared from 1 μg aliquots of RNA using a High Capacity cDNA Reverse Transcription Kit (Qiagen, Crawley, UK) and diluted 1 in 20 in DEPC-treated water to give a working stock. For quantitative real-time PCR, 12.5 ng aliquots of cDNA were amplified in triplicate on an ABI 7500 real-time PCR system using SYBR® Green primers FastStart SYBR Green Master Mix (Rox) (Roche Diagnostics Ltd, West Sussex, U.K), and primers for human genes or endogenous control GAPDH. The endogenous control GAPDH, had previously been verified as being suitable reference genes for the cell line used using the geNorm assay (Primerdesign, Southampton, UK). Relative quantification was carried out and calibrated to control samples where appropriate. Data was analysed using the standard software for the ABI 7500 real-time PCR system. All primers used for SYBR® Green RQ-PCR were designed in Primer-BLAST, a specific primer finding tool (http://www.ncbi.nlm.nih.gov/tools/primer-blast) using sequences imported from NCBI Gene. Primers were ordered from Integrated DNA Technologies (IDT) and diluted using the relevant volume of nuclease-free water. Primers were assessed by melt curve analysis following RQ-PCR. Primer sequences are listed in the Supplementary Materials (Supplementary Table 2).

### Protein extraction and quantification

Cells were pelleted by centrifugation at 200 X g for 5 minutes and resuspended in 200 μl RIPA buffer with a protease inhibitor cocktail (Sigma-Aldrich Company Ltd., Germany). Lysates were incubated on ice for 30 minutes before centrifuging at 10,000 X g for 20 minutes to remove cell debris. Protein concentrations were determined using the BCA Protein Assay Reagent (Pierce, Rockford, IL). Thirty to 50 μg of protein lysates were resolved by SDS-polyacrylamide gel (12%). The gels were electroblotted onto nitrocellulose membranes (Hybond-P, Amersham, GE Healthcare). Antibody staining was performed with a chemiluminescence detection system (Supersignal; Pierce). PARP (14-6666;eBioscience, Cheshire, UK), SHH (ab53281; Abcam, Cambridge, UK), hyperacetylated histone H3 (#9715; Cell Signalling, Leiden, NL), H3K9Ac (#9649; Cell Signalling, Leiden, NL) and acetylated α-tubulin (#3971; Cell Signalling, Leiden, NL) monoclonal antibodies were used in conjunction with a horseradish peroxidase (HRP)-conjugated sheep anti-mouse or anti-rabbit secondary antibody (P0447 and P0448; Dako, Denmark). Equal loading was assessed using a β-actin mouse monoclonal primary antibody (Sigma, Poole, Dorset, UK).

### Flow cytometry

Cells were fixed by the addition of 1 ml of ice cold 70% ethanol to cell pellets, and resuspended by pipetting up and down. Cells were pelleted by centrifugation at 200 X g for 5 minutes, and resuspended in PI (propidium iodide (40 μg/L)/RNase solution (20 μg/L)) in PBS. Samples were incubated at 37°C for 30 minutes before analysing using the BD LSR II and the BD FACSDiva Software (BD Biosciences, Oxford, UK).

### Inhibitors

The HDAC inhibitor Vorinostat and sonic hedgehog pathway inhibitor SANT-1 (Selleck Chemicals, Munich, Germany), were prepared in high purity DMSO, and stored in aliquots at −80°C until required.

### Chromatin immunoprecipitation and deep sequencing (ChIP-SEQ)

Following 24 hour treatment with either 1 μM Vorinostat or control conditions, 2×10^6^ OCI-AML3 cells were washed and pelleted. ChIP was achieved using the MAGnify™ Chromatin Immunoprecipitation System (Thermo Fisher Scientific, Paisley, UK) following the manufacturer's guidelines, with additional optimisation where needed. In brief, chromatin was crosslinked in a final concentration of 1 % formaldehyde for 10 minutes recommended by the protocol, and resuspended to 2 million cells per 500 μl of lysis buffer into 1.5 ml TPX microtubes (Diagenode, Liège, Belgium). The Bioruptor® UCD-200 was used to generate 100-300 bp fragments. To optimise the shearing, 1.5 ml TPX microtubes of prepared chromatin high was sonicated for 15 seconds ON/OFF for three 5 minute intervals, followed by a 7-minute interval (5 min, 5 min, 5 min, 7 min). Ice was replenished between each interval. Before proceeding with the protocol, the sonication of fragments to the appropriate size was evaluated on a 2% DNA gel. Sonicated chromatin was diluted to 2×10^6^ cells/ml. The IP was performed by binding 10 μg of antibody, H3K9Ac (49-1009), Pol II (9-1033) or IgG controls (Thermo Fisher Scientific, Paisley, UK) to Dynabeads® (Thermo Fisher Scientific, Paisley, UK), and incubating overnight at 4°C with 100 μl (2×10^5^cells). Samples and controls were reverse crosslinked with Proteinase K and purified with DNA Purification Magnetic Beads before eluting in 50 μl of elution buffer. Ion ChIP-SEQ libraries were prepared and adapters added using the Ion Xpress Fragment Kit (Thermo Fisher Scientific, Paisley, UK). After the library preparation, the quality of the library was assessed by the Agilent 2100 Bioanalyzer™ instrument with Agilent High Sensitivity DNA Kit (Agilent Technologies, Cheshire, UK). Libraries were diluted with Low TE buffer to a final concentration of 15 pM. Ion Sphere Particles were prepared with the template positive libraries using the Ion OneTouch™ System. After setup and initialisation of the Ion OneTouch™ System, an amplification solution was prepared and installed, and ISPs enriched as per the Ion OneTouch™ 200 Template Kit v2. Following the removal and washing of the enriched ISPs, the ISPs were stored at −20°C until sequencing. The sequencing of the prepared ISPs, using the PGM™ platform, was performed as per the Ion Sequencing Kit User Guide v2.0, along with the relevant reagents in the Ion PGM™ Supplies Kit, Ion Sequencing Reagents Kit and Ion PGM™ Reagents Kit on the Ion 318™ Chips (Thermo Fisher Scientific, Paisley, UK).

The FASTQ files from the Torrent Server were sorted and aligned using Bowtie2 [24, 25] resulting in a SAM file (Sequence Alignment Map) which was converted to a BAM file (Binary Alignment Map) whilst the quality of reads was assessed using the MAPQ (MAPping Quality). Using BEDtools, files were converted from BAM files to BED files [54] for viewing in the Integrated Genomics Viewer (IGV) (available from http://www.broadinstitute.org/igv/) [55, 56].

Enriched ChIP regions were identified using the peak finder SICER (window size of 400 bp and a gap size of 1200 bp) [26]. The peaks identified were then mapped to the nearest gene via Entrez ID using HOMER, (Hypergeometric Optimization of Motif EnRichment) [57]. The HOMER files were imported into Partek Genomics Suite 6.0 whereby Gene Ontology analysis could be carried out to determine the functionality of the genes enriched.

### Xenografts

OCI-AML3 cells were seeded the day before transfection at 5×10^6^ cells/10 cm dish and grown to approximately 70-80% confluency. Transfection of 293T cells was performed using Turbofect (Thermo Scientific, Massachusetts, USA) with a molar of 1:3:6 ratio of VSV-G envelope vector pMD2.G, packaging vector psPAX2, and firefly luciferase vector pSLIEW (kindly provided by Prof Olaf Heidenreich). Viral supernatant was harvested and filtered 72 hours after transfection. A volume of 500μl filtered pSLIEW virus was added to 2×10^5^ cells in 200 μl medium with 8 μg/ml Polybrene (Sigma Aldrich) and incubated for 48 hours before selection in neomycin. Validation of OCI-AML3 transduction was performed using Bruker *In Vivo* Xtreme imaging system before cells were treated and transplanted.

Cells were treated *in vitro* with control, each single agent and 1 μM Vorinostat plus 2.5 μM SANT-1. As 2.5 μM SANT-1 showed the greatest synergy *in vitro*, this was brought forward for our xenograft study. A total of 1.5×10^6^ cells per mouse were counted via Trypan Blue staining, resuspended in PBS and transplanted via tail vein injection into recipient NSG mice (each treatment group n=2). Mice were imaged using the Bruker *In Vivo* Xtreme imaging system three times. Mice were sacrificed at the first sign of disease and survival extension was assessed. Animal handling followed the guidelines of the UK Animals (Scientific Procedures) Act 1986 and were carried out under the Home Office License (approval granted 11th March 2014, license number PPL2760). Experimental procedures were approved by the Animal Welfare Ethical Review Board (AWERB), Queen's University Belfast.

### Statistical analysis

Unless otherwise indicated, all data is presented as the mean± s.e.m. and P values were calculated by two-tailed Student's t-test using GraphPad Prism software. Significant statistical differences (*P<0.05, **P<0.01, ***P<0.001) are indicated.

## SUPPLEMENTARY MATERIALS FIGURES AND TABLES




