# Supplementary material for: Integrated analysis of the molecular action of Vorinostat identifies epi-sensitised targets for combination therapy
Source: Oncotarget. 2017 Jul 1;8(40):67891–903. doi: 10.18632/oncotarget.18910 (PMC5620222; doi:10.18632/oncotarget.18910)
Supplement: Supplementary file 1 [file oncotarget-08-67891-s001.pdf]

Integrated analysis of the molecular action of Vorinostat identifies epi-sensitised targets for combination therapy

SUPPLEMENTARY MATERIALS

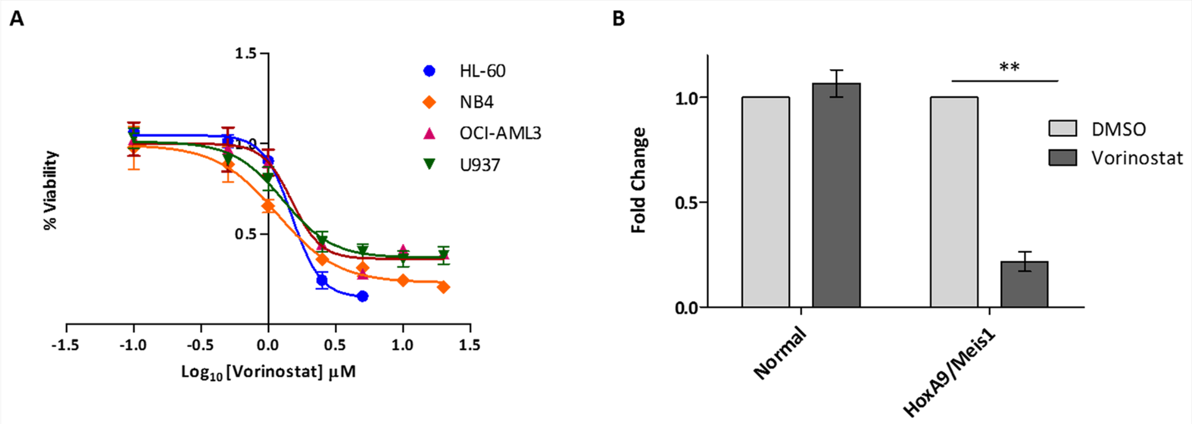

Supplementary Figure 1: Vorinostat exhibits preferential cytotoxicity in leukemic cells. **(A)** Vorinostat exhibits similar toxicity in a panel of four AML cells lines including HL-60, NB4, U937 and OCI-AML3. Despite showing potency in leukemic cells, Vorinostat spares normal cells. **(B)** Normal murine bone marrow and leukemic cells derived from a *HoxA9/Meis1* overexpressing mouse model were treated for 24 hours with 1 μM Vorinostat. Cell viability was measured using the CellTiter-Glo® assay. Data represents a technical replicate of 3. Significance, as calculated by two-way ANOVA, is denoted as * = P<0.05, ** = P<0.01 and *** = P<0.001.

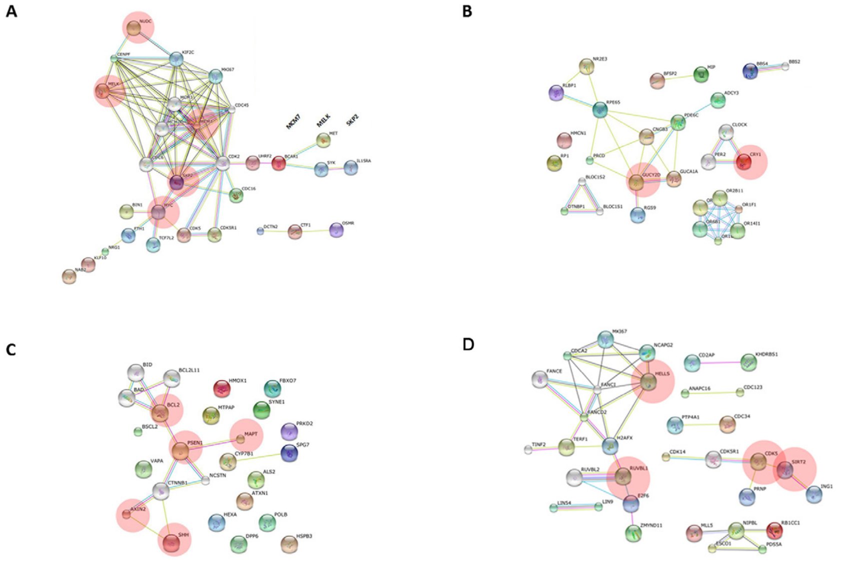

Supplementary Figure 2: Identifying the biological processes associated with changes in histone H3K9 acetylation. Gene lists associated with specific biological functions of interest, those which have features in the functional annotation of this ChIP-SEQ dataset were compiled. Gene lists were subjected to network analysis using STRING to identify significant nodes of genes associated with increased H3K9Ac following Vorinostat treatment: **(A)** cell proliferation, **(B)** metabolism, **(C)** cell death and **(D)** cell cycle. Additional nodes, shown in white, were added to further connect genes. Genes highlighted in red were selected for further validation by RQ-PCR. Data is presented as a fold change relative to the DMSO control, whereby any genes below the x-axis are down-regulated and those above, up-regulated. Experiment was carried out in a biological replicate of three. Significance, as calculated by two-way ANOVA, is denoted as * = P<0.05, ** = P<0.01 and *** = P<0.001.

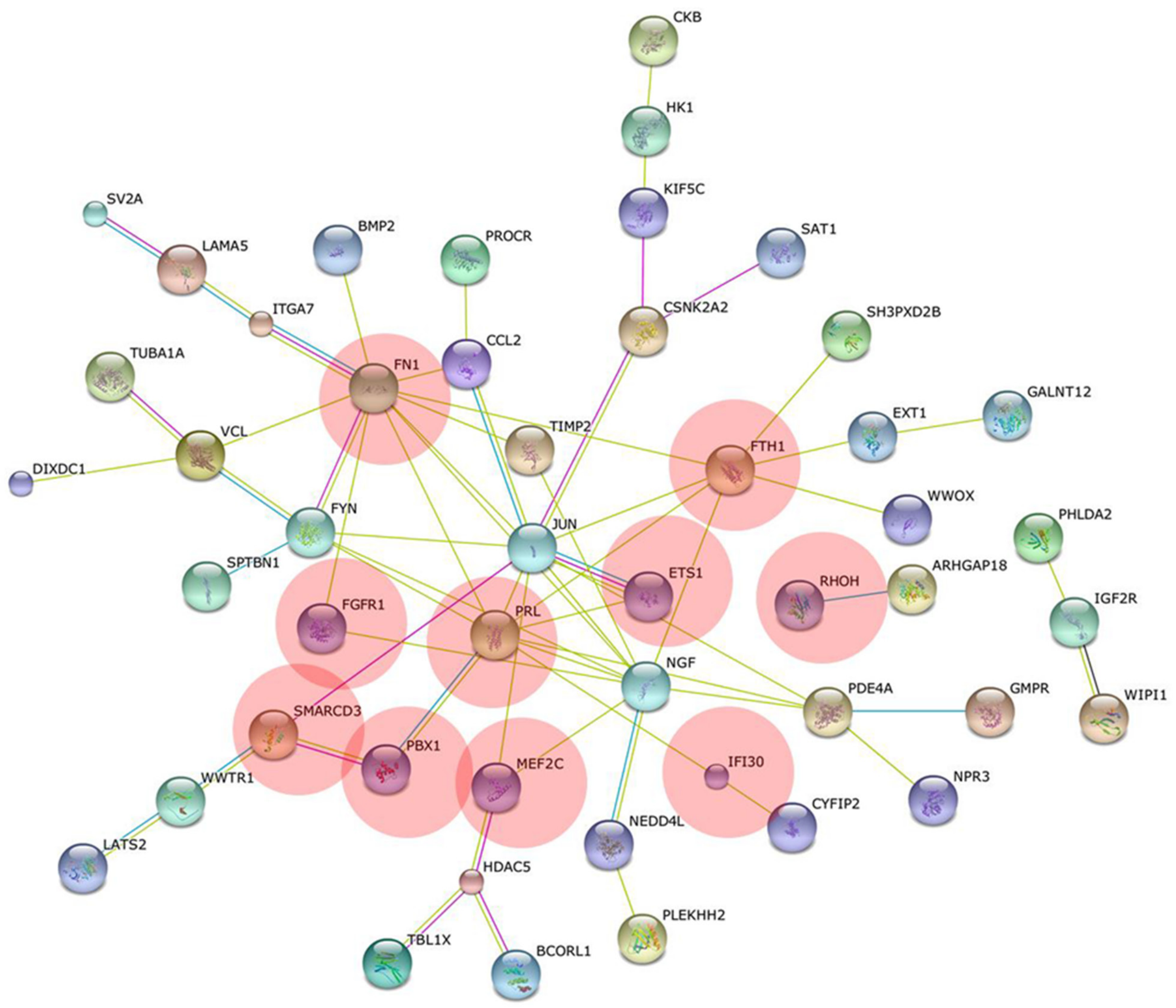

Supplementary Figure 3: Protein-protein interaction networking of genes which Vorinostat significantly altered in both gene expression and in H3K9Ac binding. Gene lists were created following the overlapping of data using BioVenn, whereby overlaps were genes which were associated with an increase in H3K9Ac/increased expression, and genes which were associated with a decrease in H3K9Ac/decreased expression. A list of 142 genes was mapped to assess protein-protein interactions using STRING. Unconnected nodes were removed, resulting in 48 networked nodes. Genes highlighted in red were randomly selected for validation by RQ-PCR.

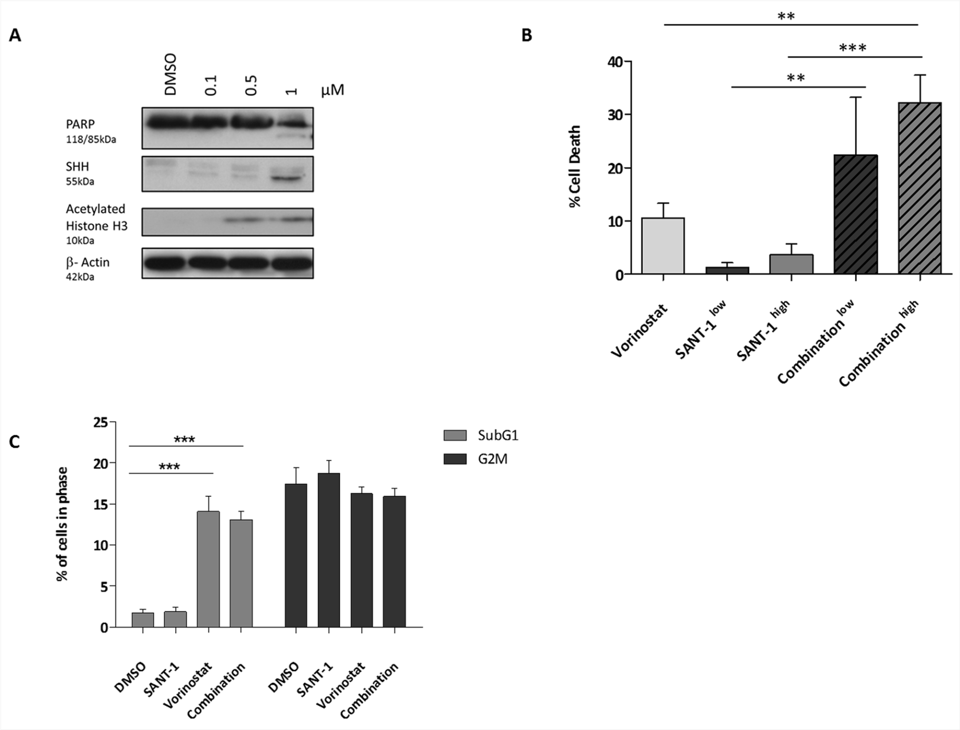

Supplementary Figure 4: The sonic hedgehog signalling pathway is a potential therapeutic target for combination therapy. (A) HL-60 cells treated with Vorinostat or DMSO for 24 hours. Equal loading was confirmed with β-Actin. **(B)** Percentage cell death of HL-60 cells was measured using the CellTiter-Glo® viability assay following 24 hours treatment with either sequential treatment of 1 µM Vorinostat with low (0.1 µM) or high dose (2.5 µM) SANT-1, or single agents. Data represents a biological replicate of 3, with data presented relative to the DMSO control (0% cell death). **(C)** Cell cycle analysis of propidium iodide stained OCI-AML3 cells in SubG1 and G2-M phases of the cell cycle following 24 hours with either DMSO vehicle control, single agents or combination treatment. Significance, as calculated by two-way ANOVA, is denoted as * = P<0.05, ** = P<0.01 and *** = P<0.001. Data represents a biological replicate of 3.

Supplementary Table 1: Genes identified as being significantly up- or downregulated following Vorinostat treatment as identified by transcriptome analysis.

See Supplementary File 1

Supplementary Table 2: RQ-PCR primer sequences

ADI1_F	GTATATGGACGACGCCCC	ADI1_R	TCATTCTCATATTTGTCAGCATCC
AXIN2_F	GTCCATGACGGACAGCAGTG	AXIN2_R	TGGGTTCTCGGGAAATGAGGT
BCL2_F	AGATTGATGGGATCGTTGCCT	BCL2_R	AGTCTACTTCCTCTGTGATGTTGT
BRD4_F	TCTGACAGCGAAGACTCCGA	BRD4_R	GGTGGTGATGATGGTGCTTCT
CDK5_F	GGTGACCTCGATCCTGAGATTG	CDK5_R	CCCAAAGAGGACATCCGGT
C-FOS_F	TTAGGACATCTGCGTCAGCAGGTT	C-FOS_R	TCTCGTGAGCATTTCGCAGTTCCT
CRY1_F	GGACTAGGTCTTCTGGCATCA	CRY1_R	ACCACTCCCTTGAGAGCAAC
CYSTM1_F	CAGACCCGACCGTTATCCAG	CYSTM1_R	TCCTGGACCCATTGGTTGTG
ETS1_F	GGCCAAAACCGTTGTTGTGTT	ETS1_R	AGAATCCTCAGCCGGCAAAT
FCGR1B_F	ATGGCACCTACCATTGCTCA	FCGR1B_R	TGGTAACTGGAGGCCTTTCAC
FGFR1_F	CACAGAGACCCACCTTCAAGC	FGFR1_R	TGGTTGGAGGTCAAGGCCAC
FN1_F	CTGGCCAGTCCTACAACCAG	FN1_R	AGTGTTTGTTCTCTGATGGTATCT
FTH1_F	CAGGTGCGCCAGAACTACCA	FTH1_R	AAAGCCACATCATCGCGGTC
GAPDH_F	GTCGCCAGCCGAGCCACATC	GAPDH_R	GGTGACCAGGCGCCCAATACG
GUCY2D_F	GTCTATGAGGGAGACAGGGTTTG	GUCY2D_R	GGTAGAGGGCCACGTTCTCA
HELLS_F	TGACGAAAATGGAACAGCAACA	HELLS_R	CCTCATAACTGGCTTCTCTTCACT
IFI30_F	CCCTCTGCAAGCGTTAGACT	IFI30_R	CAAGCGGTGCATTGGACTTC
IL8_F	TACTCCAAACCTTTCCACCCC	IL8_R	CCCAGTTTTCCTTGGGGTCC
IQGAP3_F	AAAGAACATGCCCCGGGTAG	IQGAP3_R	TATCTGAGGGGCCAATCCCA
LAMA5_F	TGCTGGACCTCATTGTGACC	LAMA5_R	GCGGCAGCCAAAATAGTCAG
LIMA1_F	AATAGCCTGGCAGTCCGTTC	LIMA1_R	ACAGGCTGTTGAACCTCACT
MAPT_F	AGTTGACCTGAGCAAGGTGAC	MAPT_R	GGACCCAATCTTCGACTGGAC
MCM7_F	CCCAGTTTGAACCTCTGGACA	MCM7_R	TCCACGTATGCTGCTGTGAT
MEF2C_F	CACTGGCTCACCCTTCTCTG	MEF2C_R	ATTGCCATACCCGTTCCCTG
MELK_F	CCAGTGCCTGAAAGAAACTCC	MELK_R	ATCCAATTCCACTGAGCGGC
MYC_F	GGACCCGCTTCTCTGAAAGG	MYC_R	TAACGTTGAGGGGCATCGTC
NUDC_F	TGCAGGAGCTTGTGAACACCTT	NUDC_R	TTGTGGTGGCTGAAAGTCTGT
PBX1_F	CACAGGTGGATACCCTTCGC	PBX1_R	CCACGTTGGAAGTCGGAAGT
PRL_F	GTGACCCTTCGAGACCTGTT	PRL_R	GGCCTTGGTAATGAACCCCC
PSEN1_F	CCTGCACCGTTGTCCTACTT	PSEN1_R	GTGCTCCTGCCGTTCTCTAT
RHOH_F	TTCTTCGGCATTCTGCAACAG	RHOH_R	TCTTCAACTGGTGTGTGAAGGAGAA
RUVBL1_F	TTGCATGACTTGGATGTGGC	RUVBL1_R	CCCCTCGAAGTTTGTCTGTG
SAT2_F	CTGCAATCATCCAATGGTCG	SAT2_R	GATTCCATTCGGGTCCATTC
SF3A1_F	ACGGACCAGGTCTCTGTCAT	SF3A1_R	CGCCATTGGCCATGTTGTAG
SHH_F	CCCCAATTACAACCCCGACA	SHH_R	GGCCAAAGCGTTCAACTTGT
SIRT2_F	GCACGGCACCTTCTACACAT	SIRT2_R	AGACGATATCAGGCTTCACCAG
SMARCD3_F	TGGCCGCGGACGAAGTT	SMARCD3_R	TTTGCTTTTCGTGGCTTTGCG
TAPBP_F	TGTTGGTTCGTGGAGGATGC	TAPBP3_R	GGTGAGGACAGTCAGTACCA
TCN1_F	GGTCTTACCTGCCCTGATGG	TCN1_R	GTGAGTCAGGAGGTGTCACAG
