# Supplementary material for: Integrated analysis of the molecular action of Vorinostat identifies epi-sensitised targets for combination therapy
Source: Oncotarget. 2017 Jul 1;8(40):67891–903. doi: 10.18632/oncotarget.18910 (PMC5620222; doi:10.18632/oncotarget.18910)
Supplement: Supplementary file 2 [file oncotarget-08-67891-s002.docx]

| ***Increasing***  IL8  TCN1  LIMA1  CYSTM1  PRL  FGFR1  PNMA2  F11R  WIPI1  VAT1L  FAM70A  USP53  TUBA1A  STC1  SAMSN1  CCDC151  FYN  MFAP2  CCL2  SRPX  FHL1  SAT1  GMPR  SYNPO2  AGTRAP  BCL2A1  PFKM  PROCR  EXT1  RIN2  SYT11  PHLDA2  PFN2  Hs.659940  FN1  CD59  RAB31  S100A10  KIF5C  PPIC  SMARCD3  MTSS1  RHOH  IFI30 | TMEM38A  DIXDC1  ETS1  LPHN1  JUN  TP53INP2  A2LD1  SERPINB10  MXRA7  GZMA  MLLT11  H1F0  COL15A1  TMOD2  SQRDL  CDC42EP3  LOC729013  LOC283674  SYNGR1  BMP2  ARID5B  LOC100506922  JHDM1D  NPR3  ZCCHC5  CXXC5  CD46  RALGAPA2  ANKRD22  ASMTL  OXR1  WWTR1  TBL1X  CKB  KCNMB4  GRK5  RAB13  KCNK5  LAMA5  TPD52L1  HK1  IFT80  TUBA4A  PBX1  FBXO18  PREX1 | FTH1  DGKA  FAM134B  GALNT12  PLEKHH2  BCORL1  SEMA6A  EGFL8 /// PPT2 /// PPT2-EGFL8  KIF1B  NIPSNAP1  SPTBN1  HN1  FNDC3B  RFX2  STON1  PTPN14  COL1A1  TMSB4X  CYFIP2  WDR44  PEX1  PPP1R26  GLYR1 /// SEPT6  LATS2  CSNK2A2  FYCO1  GABARAPL1  PGPEP1  ITM2C  CD163L1  SNX10  CCPG1 /// DYX1C1-CCPG1  IGF2R  TCEAL4  MEF2C  ACP6  PLXND1  GPR157  CORO1C  ITGA7  ACRBP  ACSF2  SLC46A3  METTL9 | ECI2  LOC100507507  PVRL2  MYADM  GPR155  NIPAL3  NEDD4L  ARHGAP18  SH3PXD2B  PAPPA  MIR22 /// MIR22HG  C16orf70  C9orf89  HRH1  MERTK  FBXO15  H2AFJ  FEZ1  DDIT4  AGXT2  RPS15A  GALE  TLE1  TACSTD2  HEATR7A /// LOC100652949 /// LOC377711  TRIM9  BTN3A2  TCTEX1D1  SV2A  LY86  HDAC5  PDE4A  RASA3  TIMP2  VCL  NGF  ZNF544  CCBL1  WWOX  PAM  LOC283683 /// LOC646278  CBY1 | ***Decreasing***  ADI1  IQGAP3  FCGR1B  SF3A1  TAPBP  ING3  PHF19  MS4A6A  ZMYND11  CTPS1  NBN  CENPT  CCNA2  CLSTN2  TLN1  CAPN7  RXFP1  HYLS1  SMG9  PMF1  CNOT3  GEN1  GALNT1  KEAP1  ELANE  ENSG00000261179  GALNT7  GTSE1  IDH3A  ALDH5A1  P2RY2  ARHGAP9  NDC80  SLC25A13  ZNF318  LOC730102  NAA25  PTK2B  TTC27  ZBTB33  PCBD2  THG1L  ANLN  TOR1AIP1 | ATP13A3  SAP30L  RNFT1  GBE1  ITSN2  DIS3L  SFMBT1  NAGA  NIPSNAP3A  CS  EPHX1  GTF3C6  INTS7  GBF1  TRMT5  AVEN  TUBGCP5  BRPF3  ATAD2B  SRD5A3  DEPDC1  EFCAB2  MCM5  OTUB1  BRCA1  SMC1A  CDK3 /// TEN1-CDK3  SLC10A4  PSRC1  APOBR  XPOT  EIF3M  LANCL2  HJURP  STIL  C7orf55  LILRB4  METTL21D  YWHAE  ZNF689  IDH2  DDX11 /// DDX12P /// LOC642846  UNG  DNAJC9  MDC1 | NCAPG2  PDS5B  CENPH  SMAD4  SRRM1  SULT1A2  OGDH  EXO1  TEP1  PATZ1  MCM10  CTNND1 /// TMX2-CTNND1  FAM122B  EHD1  NFYC  GINS2  IL12RB1  PGK1  SDAD1  SIRPB1  PTRHD1  HMGN2  ACTL6A  DPP3  SLX1A-SULT1A3 /// SLX1B-SULT1A4 /// SULT1A3 /// SULT1A4  ALDH6A1  SSRP1  CIZ1  ACSL5  CALCOCO2  TTK  VAMP8  BCLAF1  CEP78  SEPHS1  DNAJA4  ILF3  COPS7A  FAM178A  KPNA4  SERP1  TXNDC15  SYPL1 | CPSF7  DHRS9  EPC1  NCOA5  ZNF259 /// ZNF259P1 |
| --- | --- | --- | --- | --- | --- | --- | --- |
